# Accuracy comparison of various quantitative [^99m^Tc]Tc-DPD SPECT/CT reconstruction techniques in patients with symptomatic hip and knee joint prostheses

**DOI:** 10.1186/s13550-021-00794-7

**Published:** 2021-06-14

**Authors:** Martin Braun, Michal Cachovan, Felix Kaul, Federico Caobelli, Markus Bäumer, A. Hans Vija, Geert Pagenstert, Damian Wild, Martin Kretzschmar

**Affiliations:** 1 Division of Nuclear Medicine, University Hospital Basel, Basel, Switzerland; 2 Siemens Healthcare GmbH, Molecular Imaging, Forchheim, Germany; 3 Siemens Medical Solutions USA, Inc., Molecular Imaging, Hoffman Estates, USA; 4 Department of Orthopedic Surgery, University Hospital Basel, Basel, Switzerland

**Keywords:** SPECT/CT, Quantification, Arthroplasty, Aseptic loosening

## Abstract

**Background:**

There is a need for better diagnostic tools that identify loose total hip and knee arthroplasties. Here, we present the accuracy of different ^99m^Tc-dicarboxypropandiphosphate ([^99m^Tc]Tc-DPD) SPECT/CT quantification tools for the detection of loose prostheses in patients with painful hip and knee arthroplasties.

**Methods:**

Quantitative reconstruction of mineral phase SPECT data was performed using Siemens xSPECT-Quant and xSPECT-Bone, with and without metal artefact reduction (iMAR) of CT-data. Quantitative data (SUVmax values) were compared to intraoperative diagnosis or clinical outcome after at least 1 year as standard of comparison. Cut-off values and accuracies were calculated using receiver operator characteristics. Accuracy of uptake quantification was compared to the accuracy of visual SPECT/CT readings, blinded for the quantitative data and clinical outcome.

**Results:**

In this prospective study, 30 consecutive patients with 33 symptomatic hip and knee prostheses underwent [^99m^Tc]Tc-DPD SPECT/CT. Ten arthroplasties were diagnosed loose and 23 stable. Mean-SUVmax was significantly higher around loose prostheses compared to stable prostheses, regardless of the quantification method (*P* = 0.0025–0.0001). Quantification with xSPECT-Bone-iMAR showed the highest accuracy (93.9% [95% CI 79.6–100%]) which was significantly higher compared to xSPECT-Quant-iMAR (81.8% [67.5–96.1%], *P* = 0.04) and xSPECT-Quant without iMAR (77.4% [62.4–92.4%], *P* = 0.02). Accuracies of clinical reading were non-significantly lower compared to quantitative measures (84.8% [70.6–99.1%] (senior) and 81.5% [67.5–96.1%] (trainee)).

**Conclusion:**

Quantification with [^99m^Tc]Tc-DPD xSPECT-Bone-iMAR discriminates best between loose and stable prostheses of all evaluated methods. The overall high accuracy of different quantitative measures underlines the potential of [^99m^Tc]Tc-DPD-quantification as a biomarker and demands further prospective evaluation in a larger number of prosthesis.

## Background

The prevalence of total hip and knee arthroplasties (THA, TKA) has been constantly increasing over the last decades [1]. As a result, the number of dysfunctional or loose prostheses needing revision surgery is increasing. Reported rates of prosthetic loosening for THA and TKA range from 2% after 5 years to 8% after 15 years and of 2.3% after 5 years to 7.1% after 15 years, respectively [2]. Aseptic loosening is the most common reason for revision operations of THA (40%) and TKA (36%) [3].

In clinical routine diagnostic work up of symptomatic joint prosthesis is based on a combination of clinical examination and imaging studies. Plain radiographs are the standard imaging method for follow-up of joint replacements [4]. They are used to assess the integration, alignment and integrity of implants. However, the interrater agreement of radiographic assessment of prosthetic loosening is low [5] as is the sensitivity for the detection of periprosthetic osteolysis [6]. Recent advances of metal artefact reduction in MR imaging allow the visualization and assessment of the bone-implant interface when implants with low susceptibility for metal artefacts such as titanium alloy-based implants are used. However, MR findings such as bone marrow oedema or periosteal reaction appear also but less frequent in asymptomatic prosthetic joints limiting the specificity of this imaging modality [7].

A functional imaging modality is planar bone scintigraphy or 3D-SPECT imaging of bone remodelling that uses technetium-labelled diphosphonates, in our study ^99m^Tc-Dicarboxypropandiphosphate (DPD), to target immature bone matrix (osteoid). An increased tracer uptake in bone indicates higher osteoid content of bone matrix and is therefore indicative for an increased osteoblastic activity, for example, due to mechanical stress of a loose prostheses. This technique is used as a second line diagnostic imaging method for the evaluation of painful hip and knee joint prosthesis since the 1970s with diverging reports of diagnostic accuracy [8–12]. Hybrid-scanners combining SPECT and computer tomography (CT) are used for more than 15 years. With its ability to directly correlate functional information with structural changes in morphological imaging data, this new technique is more and more replacing conventional planar scintigraphy, also for this particular diagnostic question of aseptic prosthetic loosening [13, 14]. The main focus of the diagnostic criteria of prosthetic loosening in SPECT/CT lies in the distribution of elevated bone metabolism around the prosthetic implant. Only few studies investigated the diagnostic value of uptake intensity of periprosthetic bone [15–17] with promising results although only relative semiquantitative uptake measures were used. Recently, a new generation of SPECT/CT scanners and reconstruction algorithms of SPECT data was introduced that allows the absolute quantification of tracer uptake [18] in clinical routine. Similar to positron emission tomography (PET), quantitative uptake measures can be normalized and expressed in standardized uptake values (SUV) which potentially allows the discrimination between stable and loose prostheses. The value of absolute uptake quantification of bone SPECT/CT was already investigated for several applications in musculoskeletal and oncological imaging [19–22]. To the best of our knowledge, no published data exist analysing the diagnostic value of truly quantitative measures of bone metabolism around prosthetic joint implants for the detection of aseptic loosening.

Therefore, we compared the diagnostic accuracies of different quantitative SPECT reconstruction algorithms (xSPECT Quant and xSPECT Bone) obtained with a Siemens Symbia Intevo T16 System [23, 24] with and without iterative metal artefact reduction (iMAR) in order to detect aseptic implant loosening of patients with symptomatic hip and knee joint prosthesis. Standard of comparison was surgical diagnosis or follow-up observation over a period of at least 1 year.

## Materials and methods

### Patient cohort

For this prospective single-centre study, patients with suspicion of aseptic loosening of knee or hip joint arthroplasties were consecutively enrolled between 03/2015 and 04/2018 just before the planned bone SPECT/CT scan. Only those patients were included who were managed by our orthopaedic surgeons in order to facilitate the collection of follow-up data. The following inclusion criteria applied: Patient with painful total hip arthroplasty (THA), total knee arthroplasty (TKA) or unicondylar knee arthroplasty (UKA), age older than 18 years and informed written consent.

This study was approved by the regional scientific ethics committee (EKNZ 2015–356), and all procedures in this study were performed in concordance with the Helsinki declaration and its later amendments. Informed consent was obtained from all participants included in the study.

### Image acquisition

We used a Symbia Intevo SPECT/CT scanner (Siemens Healthineers, Erlangen,) with a LEHR collimator. SPECT acquisition followed 3 h after cubital intravenous injection of 686 ± 25.4 MBq ^99m^Tc-Dicarboxypropandiphosphate (DPD). Imaging parameters for SPECT were: matrix size 256 × 256, 5.625° angular resolution in 32 steps with an acquisition time of 35 s (knee) and 30 s (hip) per step, low energy high-resolution collimator. CT scan parameters were: 130 kV, 130–160 mAs, collimation 16 × 0.6 mm, reconstruction increment 0.8 mm.

For quantitative imaging, the SPECT component was calibrated once per month using a 3%—National Institute of Standards and Technology (NIST) traceable ^57^Co source (Calibrated Sensitivity Source (CSS)).

### Image reconstruction

SPECT data were reconstructed with Flash3D with attenuation and scatter correction (Siemens, Germany) as the standard reconstruction for clinical use. Quantitative uptake maps were reconstructed with xSPECT Quant and xSPECT Bone (both Siemens, Germany), and include attenuation and scatter correction and standardized calibration for absolute quantification. xSPECT Bone uses the CT information to provide images with improved tissue boundary resolution. It uses a zone map derived from the CT data to segment the anatomical image into five specific tissue classes “zones”: cortical bone, spongiosa, soft tissue, fat tissue and air [23, 24]. For attenuation correction, CT density data with and without metal artefact reduction [25, 26] were used.

Planar scintigraphies were obtained with a triple phase protocol using the same SPECT/CT scanner system: perfusion phase immediately after tracer injection, blood pool phase ~ 3 min post-injection and delayed phase ~ 3 h post-injection.

### Image analysis

Quantitative assessment of SPECT/CT data was done by one nuclear medicine physician with 9 years of experience (MB) and one specialist in musculoskeletal radiology and nuclear medicine with 17 years of experience (MK) who were blinded from clinical information. Image data were displayed in multiplanar reconstructions (MPR) and maximum intensity projections (MIP) and analysed with a dedicated software (syngo MI Applications version VB10A, Volumetric Analysis version 10.0.1408.2901, Siemens Medical Solutions USA, Inc. and Toshiba Corp.)

**Fig. 1:**
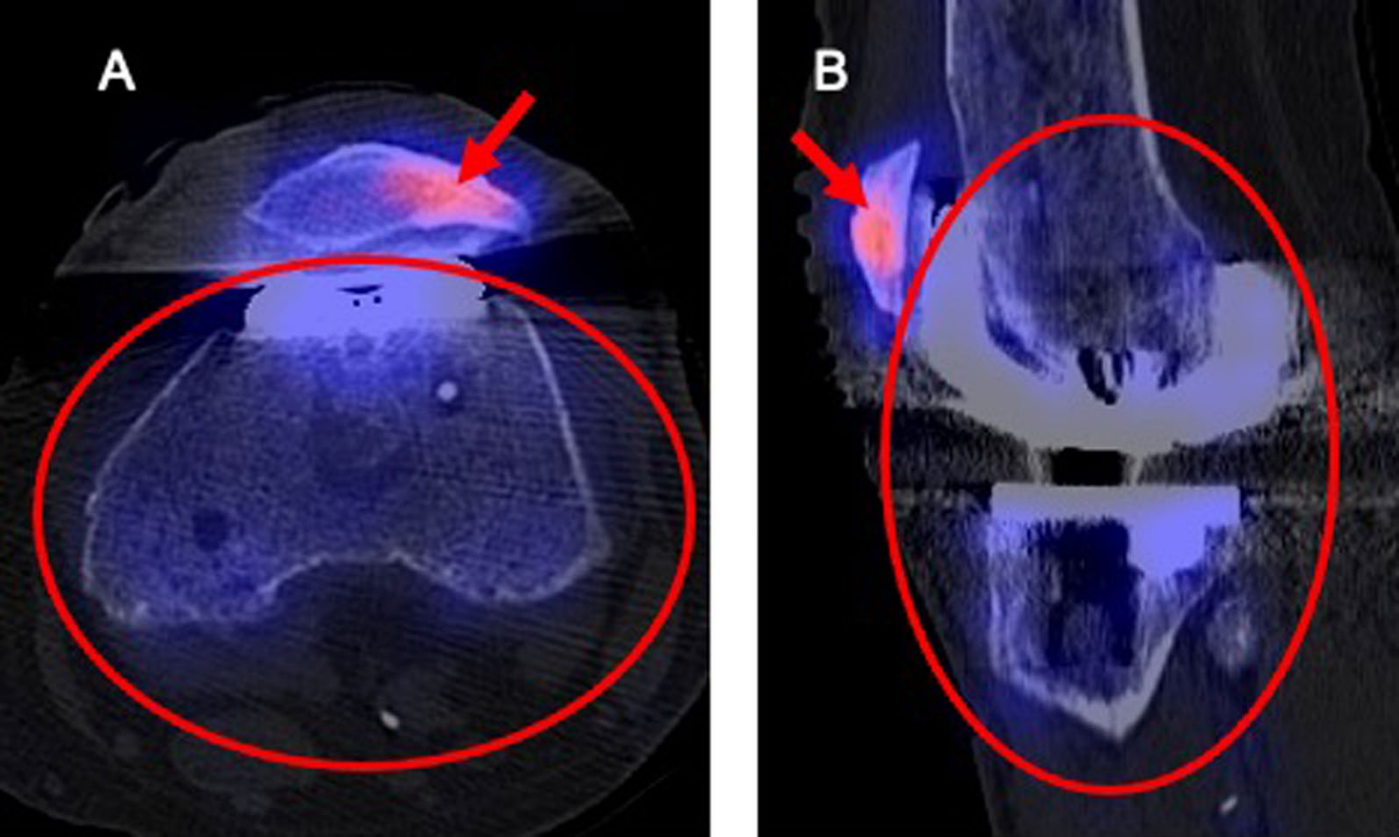
Illustration of VOI placement in a patient with a painful knee arthroplasty due to cartilage lesions of the patella. Uptake of the subchondral bone in the lateral facet of the patella (red arrow) was excluded from the VOI that included both stable components of the arthroplasty

For quantitative uptake measurement, an ellipsoid volume of interest (VOI) was drawn into the fused images that covered the whole joint, including all articulating compounds. Specific care was taken to assure that foci of uptake not associated with the bone-prosthetic interface were excluded from the VOI (i.e. osteophytes n = 4, patellar osteoarthritis n = 4, other osteoarthritis n = 1, associated with osteosynthetic material after fracture n = 1, heterotopic ossification n = 1) (Fig. 1). Uptake intensity was expressed in maximum standardized uptake values (SUV max) which is the measured activity concentration of tissue normalized by the injected activity and the patient weight.

### Visual analysis

**Fig. 2:**
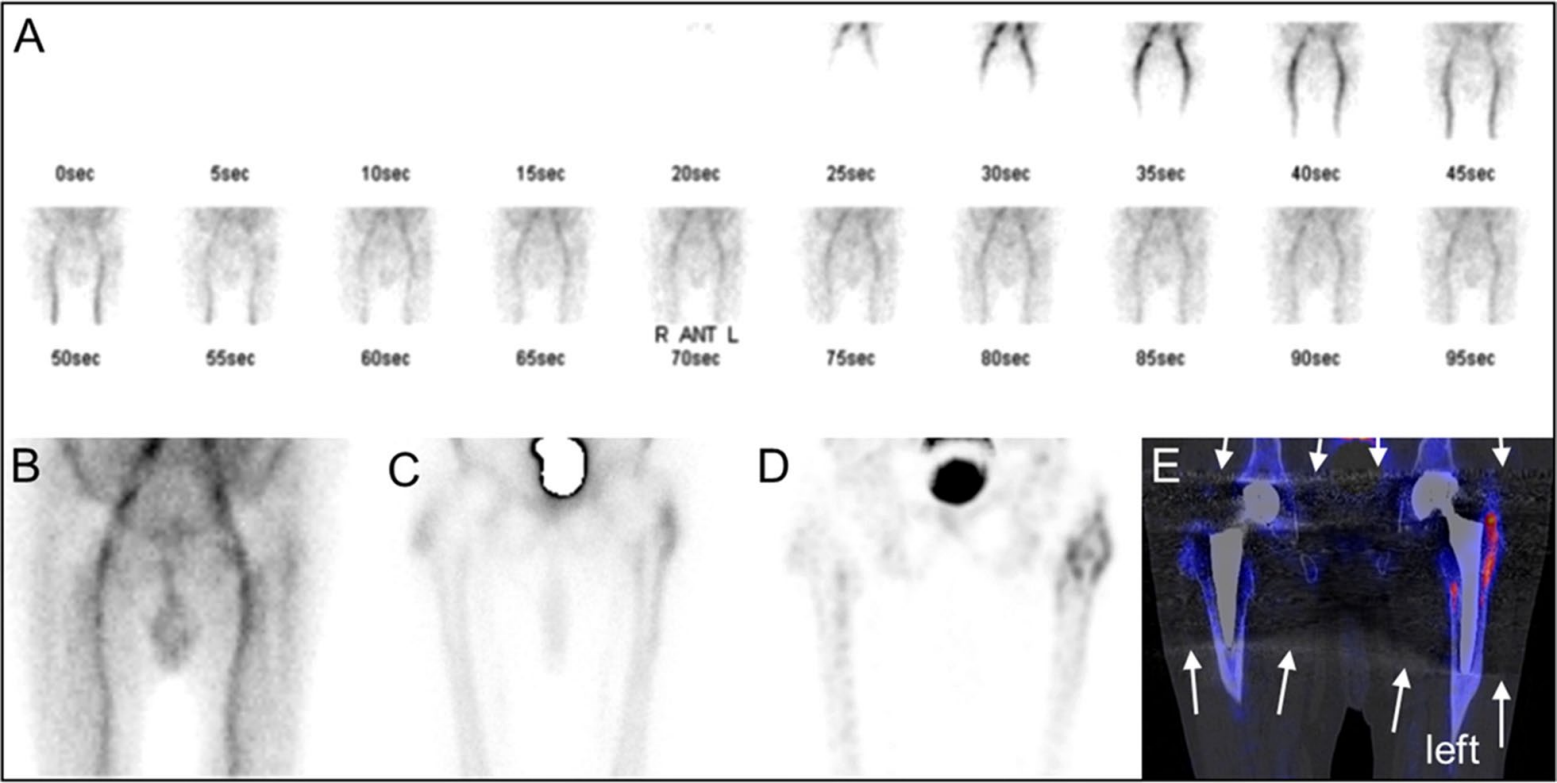
Example of a patient (male, 71 years) with bilateral hip joint replacements (1995 right, 1998 left) and chronic pain in the area of the left hip. Visual analysis was based on planar triple phase bone scan including **a** flow phase, **b** blood pool phase and **c** delayed phase as well as **d** SPECT Flash 3D maximum intensity projection and **e** SPECT Flash 3D in combination with CT. Arrows show metal artefacts. The left prosthesis stem was considered loose by visual assessment

Visual reading of SPECT/CT images was performed by one experienced and one trainee nuclear medicine physician with 7 years (FK) and 1 year (FC) of experience in musculoskeletal SPECT/CT reading. Both readers were unaware for clinical and imaging information except the fact that there was suspicion of prosthetic loosening in at least one of the displayed prostheses. SPECT/CT images as provided for clinical routine diagnosis were assessed on a clinical PACS reading platform (Centricity, GE healthcare, Chicago, USA). Diagnosis was based on visual assessment of uptake intensity, distribution of tracer uptake around the prosthesis on planar triple phase bone scans and SPECT Flash3D in combination with CT (CT without iMAR), see Fig. 2. Readers had to make a definitive diagnose (loose or stable) regardless of the level of diagnostic confidence.

### Standard for comparison

Standard of comparison was intraoperative diagnosis after revision of arthroplasty or follow-up of at least 1 year after SPECT/CT examination if no operative revision was performed.

A prosthesis was considered to be stable in cases where symptoms relieved and no radiological signs of loosening developed or if a diagnosis other than prosthetic loosening was established during the follow-up period, e.g. trochanteric bursitis, mechanical irritation of the iliotibial tract, gluteal tendon tear, scar pain, retropatellar osteoarthritis, PCL insufficiency a.o.

Prosthetic loosening was diagnosed non-surgically in cases where surgery was not feasible due to the medical conditions of the patient or if the patient did not wish to undergo surgery. In these cases, diagnosis was confirmed by an orthopaedic surgeon using clinical and radiological signs such as progressive lucencies or migration of implant over time. Only tests revealing consistency between imaging, surgery or clinical follow-up (positivity for loose prosthesis) were considered as true positive.

### Statistical analysis

The Shapiro–Wilk test was used to test the normal distribution of all SUV data. Afterwards, differences in SUVmax values of loose and stable prostheses were compared with the Kruskal–Wallis test. A multivariate data analysis (JMP version 16, SAS Institute, Cary, NC, USA) was performed in order to calculate the impact of time interval between arthroplasty implantation and study imaging as well as type of arthroplasty (cemented vs. uncemented) using SUV max values as outcome variable and prosthetic integrity (“loose/stable”), type of arthroplasty (“cemented/uncemented”), and interval between surgery and study imaging (“time interval”) as covariates.

Cut-off values of periprosthetic uptake were calculated with receiver operating characteristic (GraphPad, San Diego, USA) aiming at an optimization of accuracy (highest accuracy) for the detection of loose prostheses. Pairwise comparisons of diagnostic measures were made between all quantification methods and the two readers separately using the McNemar’s test. *P* values < 0.05% were considered significant.

## Results

**Fig. 3:**
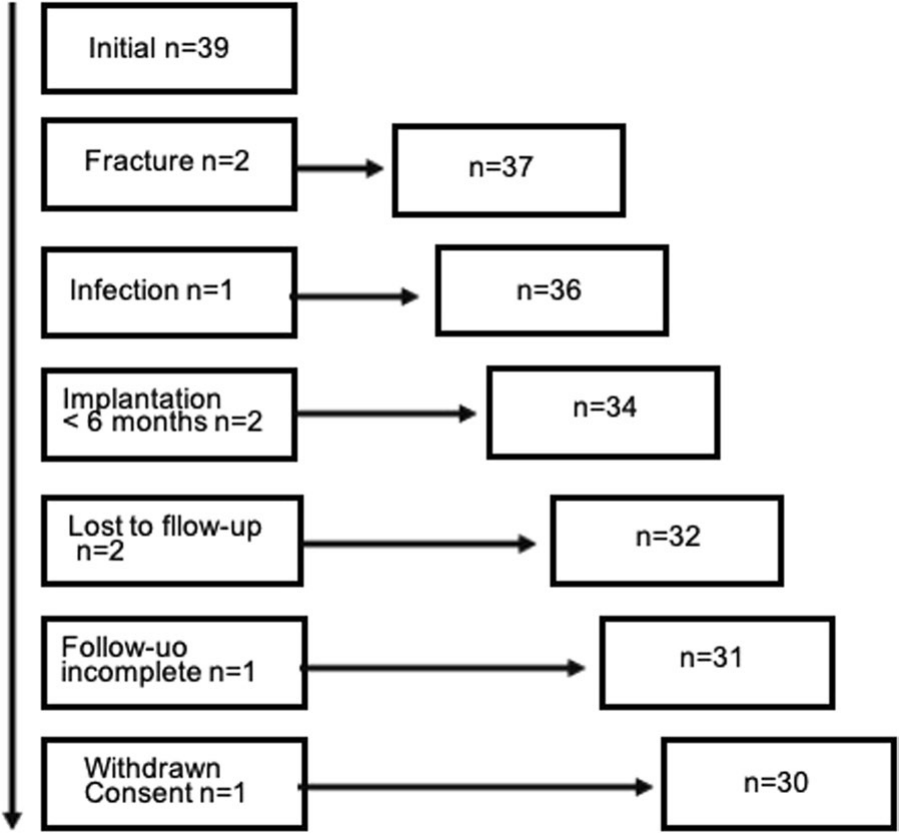
Flow diagram of the patient selection from initial cohort (*n* = 39) of referred symptomatic patients to the final cohort (*n* = 30) after application of exclusion criteria

In total, 39 patients were screened. However, 9 patients had to be excluded due to periprosthetic fractures (*n* = 2), joint infection (i.e. microbiologically proven periprosthetic infection within 1 month after SPECT/CT, *n* = 1), implantation of arthroplasty < 6 months prior to the SPECT/CT referral (*n* = 2), lost to follow-up during the study (*n* = 2), incomplete follow-up data *n* = 1), and withdrawal of consent (*n* = 1) (Fig. 3). The remaining 30 patients consisted of 17 females and 13 males with a mean age of 71 years. Mean age of prosthesis was 11.6 years. The 30 patients had altogether 44 arthroplasties (Table 1). Fourteen patients had bilateral arthroplasties (7 THA, 7 TKA), and three patients had bilateral suspicion of loosening (3 TKA).

**Table 1:** Baseline demographic and clinical characteristics

	Patients (*n* = 30)
Sex	Female 17, male 13
Age	71 (50–91) years
Weight	79 (53–114) kg
THA	20
TKA	22
UKA	2
Age of prosthesis	11.6 (1.2–28.2) years

Data are number or mean (range)

THA, total hip arthroplasty; TKA, total knee arthroplasty; UKA, unicondylar knee arthroplasty

Thirty-three of the 44 prosthesis were symptomatic and suspicious for being loose. Of the 33 symptomatic arthroplasties, loosening was confirmed for 10 arthroplasties. Of these, 3 arthroplasties were diagnosed intraoperatively. The surgical diagnoses for confirmed loose arthroplasties were: “aseptic loosening of patellofemoral compound” (1 TKA), “aseptic loosening of femoral shaft” (1 THA) and “aseptic loosening of acetabular cup” (1 THA). Loosening of 7 arthroplasties was diagnosed by follow-up.

Loosening was ruled out for 23 arthroplasties. Of these, 9 were confirmed intraoperatively by finding other diagnosis than loosening. For 14 arthroplasties, loosening was ruled out by follow-up.

The remaining 11 arthroplasties were asymptomatic prostheses in patients with bilateral arthroplasty and were included in the analysis of SUVmax measurements as normal control arthroplasties.

Twenty-three arthroplasties were cemented (13 THA, 8 TKA, 2 UKA), of which 6 were loosened prostheses (3 THA, 3 TKA).

### Quantitative [^99m^Tc]Tc-DPD uptake measurements

**Fig. 4:**
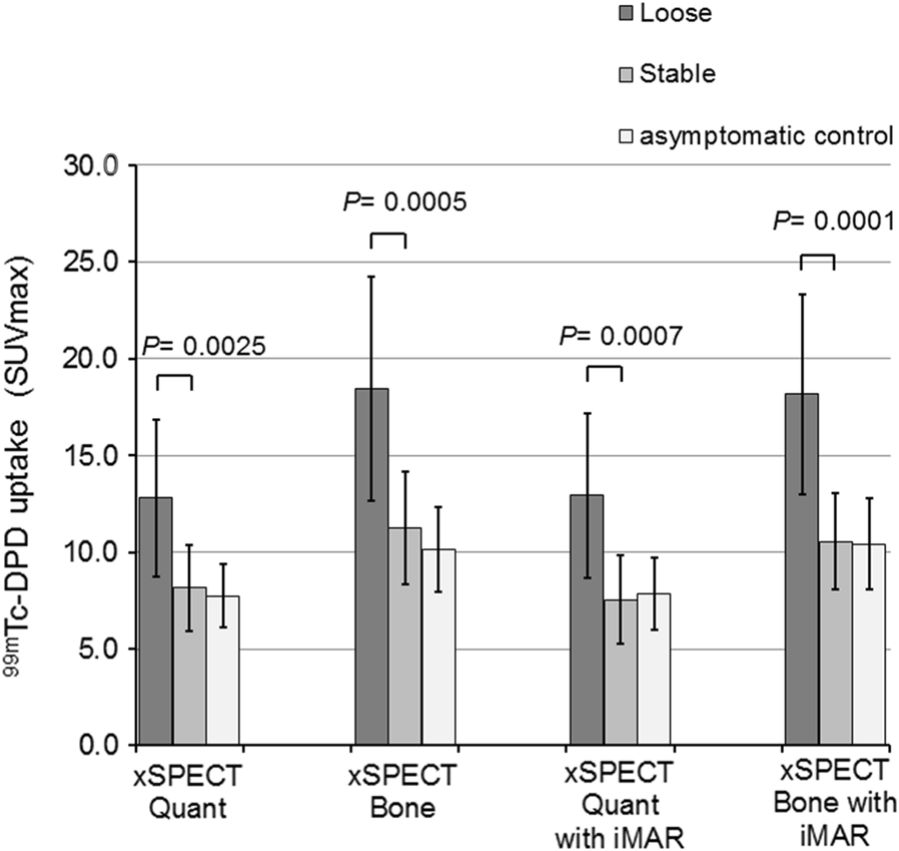
Comparison of SUVmax values between loose, stable and asymptomatic control arthroplasties. Values are expressed as mean ± SD. *P* values for comparison of loose versus stable prostheses

Compared to lose joint prostheses, stable prostheses showed significant lower mean SUVmax values (12.79–18.45 versus 7.55–11.25) independent of the SPECT quantification method (*P* = 0.0025–0.0001) (Fig. 4 and Table 2). The difference of [^99m^Tc]Tc-DPD uptake (∆ mean SUVmax ± SD) between loose and stable prostheses was lower with xSPECT Quant than with xSPECT Bone (4.63 ± 1.37 versus 7.21 ± 1.93). The combination of xSPECT Bone with iMAR further increased the difference of [^99m^Tc]Tc-DPD uptake (∆ mean SUVmax ± SD) between loose and stable prostheses (7.61 ± 1.71) resulting in the highest *P* value (*P* = 0.0001).

**Table 2:** SUVmax values of different SPECT quantification in loose and stable prostheses

	Integrity of prosthesis				Difference	SD	Kruskal–Wallis
	Loose	SD	Stable	SD			
xSPECT Quant	12.79 (9.89–15.7)	4.05	8.15 (7.14–9.2)	2.22	4.63 (1.6–7.6)	1.37	*P* = 0.0025
xSPECT Bone	18.45 (14.3–22.6)	5.77	11.25 (10.0–12.5)	2.93	7.21 (2.9–11.4)	1.93	*P* = 0.0005
xSPECT Quant iMAR	12.93 (9.9–15.9)	4.24	7.55 (6.5–8.5)	2.31	5.38 (2.3–8.5)	1.42	*P* = 0.0007
xSPECT Bone iMAR	18.16 (14.5–21.9)	5.17	10.55 (9.5–11.6)	2.49	7.61 (3.8–11.4)	1.71	*P* = 0.0001

Numbers are mean SUV max (95% confidence interval)

*P values for comparison of loose versus stable prosthesis, *n* = 10 loose prosthesis and *n* = 23 stable prosthesis

Across all prostheses (loose, stable and control) periprosthetic [^99m^Tc]Tc-DPD uptake (mean SUVmax ± SD) was significantly lower with xSPECT Quant compared to xSPECT Bone (9.14 ± 3.31 versus 12.61 ± 4.79), *P* < 0.0001 (Table 2). Iterative metal artefact reduction slightly decreased mean SUVmax values of both xSPECT Quant and xSPECT Bone; however, the difference was not significantly different (*P* = 0.73 and 0.68) (Table 3).

**Table 3:** SUVmax values of different SPECT quantification with and without iMAR across all prostheses

	xSPECT Quant	SD	xSPECT Bone	SD	Delta	SD	Kruskal–Wallis
Without iMAR	9.14 (8.1–10.2)	3.31	12.61 (11.2–14.1)	4.79	3.46 (1.7–5.2)	0.88	*P* < 0.0001
With iMAR	8.83 (8.1–10.2)	3.52	12.25 (10.7–13.6)	4.53	3.41 (1.7–5.1)	0.87	*P* = 0.0001
Delta	0.31 (–1.4–2.1)		0.36 (–1.4–2.1)				
Kruskal–Wallis	*P* = 0.73		*P* = 0.68				

Numbers are mean SUV max (95% confidence interval)

The impact of time interval between arthroplasty implantation and study imaging as well as type of arthroplasty (cemented vs. uncemented) was evaluated with a multiparametric model predicting [^99m^Tc]Tc-DPD uptake (mean SUVmax values). Calculations were performed for “time interval” and “prosthetic model” separately. The calculated least square means of SUVmax did not differ from the original [^99m^Tc]Tc-DPD uptake (mean SUVmax values). The effect probability of time interval and prosthetic model was *P* = 0.40 and *P* = 0.56, respectively.

### Diagnostic performance of quantitative uptake measures

**Fig. 5:**
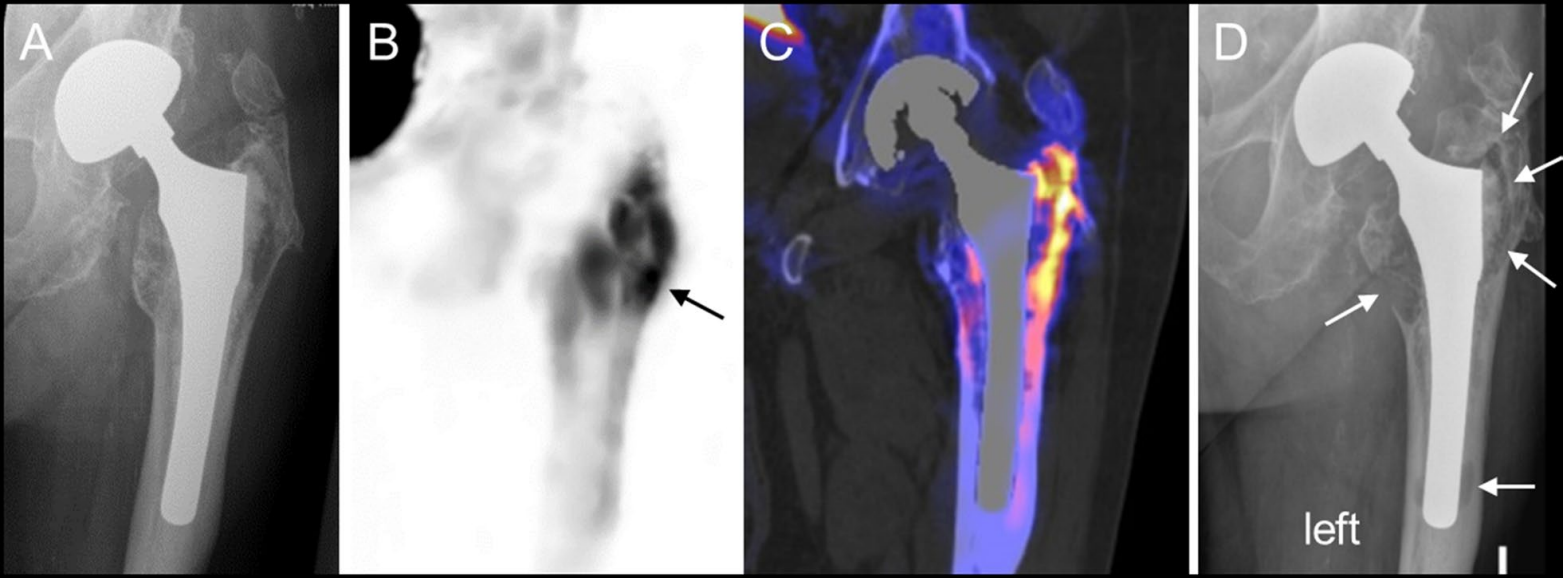
Same patient as in Fig. 1. **a** Plain radiograph at baseline without evidence for a loose prosthesis, **b** xSPECT Bone maximum intensity projection and **c** xSPECT Bone in combination with CT (CT with iMAR) at baseline as well. Periprosthetic SUVmax was 27.3 (black arrow) which was definitely above the cut-off value for a loose prosthesis (xSPECT Bone cut-off value: 14.78). Revision operation was refused. **d** Plain radiograph 2 years later showed a loose left prosthetic stem with progression of radiolucency around the femoral stem (white arrows) which correlates with pathological ^[99mTc]Tc−DPD^ uptake at baseline (**b**, **c**)

Diagnostic accuracy, sensitivity, specificity, positive and negative predictive values are given in Table 4 for all quantification methods as well as scan reading. Receiver operating characteristic (ROC) was used to calculate cut-off values of periprosthetic uptake with highest accuracy for xSPECT Quant (cut-off SUVmax 9.40 without iMAR and 9.61 with iMAR) and xSPECT Bone (cut-off SUVmax 13.78 without iMAR and SUVmax 14.78 with iMAR), see example in Fig. 5.

**Table 4:** Comparison of different xSPECT quantification as well as blinded reading in patients with suspected prosthetic loosening

xSPECT Quant	xSPECT Bone	xSPECT Quant iMAR	xSPECT Bone iMAR	xSPECT Quant	Blinded reading		Test for superiority*
					Trainee	Senior	
Cut-off SUVmax (ROC analysis)	9.40	13.87	9.61	14.78			
Accuracy	**77.4 (62.4–92.4**)	84.8 (70.6–99.1)	81.8 (67.5–96.1)	**93.9 (79.6–100)**	81.8 (67.5–96.1)	84.8 (70.6–99.1)	***P* = 0.02**
Sensitivity	90.0 (63.6–100)	90.0 (64.0–100)	90.0 (64.0–100)	80.0 (54.0–100)	60.0 (34.0–86.0)	70.0 (44.0–96.0)	*P* = 1.0
Specificity	**71.4 (53.2–89.6)**	82.6 (65.5–99.7)	78.3 (61.1–95.4)	**100.0 (82.9–100)**	91.3 (74.2–100)	91.3 (74.2–100)	***P* = 0.04**
Positive predictive value	**60.0 (38.4–81.6)**	69.2 (48.0–90.4)	64.3 (43.1–85.5)	**100.0 (74.0–100)**	75.0 (49.0–100)	77.8 (51.8–100)	***P* = 0.02**
Negative predictive value	93.8 (72.2–100)	95.0 (77.9–100)	94.7 (77.6–100)	92.0 (74.9–100)	84.0 (66.9–100)	87.5 (70.4–100)	*P* = 1.0

Diagnostic performance of quantitative uptake measures is given in percentage based on quantitative ROC analysis with respective cut-off SUVmax values or blinded reading by one trainee and one senior reader with 95% confidence intervals in brackets. Standard for comparison was surgery or clinical follow-up and follow-up imaging of at least 1 year

*P values for comparison of xSPECT Quant without iMAR versus xSPECT Bone with iMAR. There was a significant difference in the accuracy, specificity and positive predictive value between xSPECT Bone with iMAR and xSPECT Quant without iMAR (*P* = 0.04 and *P* = 0.02, respectively). All other not shown *P* values were not significantly different

Bold numbers indicate signiicant differences (level of significance *P* < 0.05)

The diagnostic accuracy was lower with xSPECT Quant (77.4%) than with xSPECT Bone (84.8%). The addition of iMAR increased accuracy for both xSPECT Quant and xSPECT Bone from 77.4 to 81.8% and from 84.8 to 93.9%, respectively. Quantification with xSPECT Bone plus iMAR showed not only the highest diagnostic accuracy (93.9%) of all evaluated quantification methods but was also significantly higher than xSPECT Quant with iMAR (81.8%, *P* = 0.04) and xSPECT Quant without iMAR (77.4%, *P* = 0.02). Accuracies of clinical reading were 81.8% (trainee) and 84.8% (senior) which was inferior to the quantification with xSPECT Bone plus iMAR but not significantly different.

## Discussion

The main findings of this study are: (1) Quantitative SPECT/CT revealed a significantly increased [^99m^Tc]Tc-DPD uptake in loosened hip and knee joint replacements compared to stable joint replacements independent on the type of prosthesis. (2) Quantification with xSPECT Bone plus iMAR showed the highest accuracy of all evaluated methods in the discrimination between loose and stable prostheses. (3) Quantitative SPECT/CT seemed to differentiate better with a higher accuracy between loose and stable prostheses than conventional visual reading.

Only few studies investigated the relative intensity of periprosthetic bone metabolism with bone scintigraphy and bone SPECT/CT referencing the periprosthetic activity with ROIs of the femur [15–17] and found a good interreader agreement for such a standardized protocol [16, 17]. To our knowledge, there is only one available study applying quantification of periprosthetic uptake for the diagnosis of aseptic loosening published 2008 by Klett et al. [15]. In that retrospective study, planar scintigraphies of 31 cemented knee joint prosthesis were used placing ROIs in the periprosthetic tibia and a normalizing control ROI in the femur. With this approach, the differentiation between loose and stable prostheses was possible with an accuracy of 94% which corresponds well to our results. However, only patients with revision surgery were included and it is likely that the results of the prior bone scan contributed to the decision for surgery, thus introducing a selection bias. In contrast, we used a prospective approach, including patients prior therapeutical considerations and followed them for at least 1 year if no surgery was performed. This prospective design makes the results of our study more meaningful for clinical application. Moreover, the direct quantification of [^99m^Tc]Tc-DPD uptake after standardized calibration of the scanner with only one VOI covering the whole prosthesis is highly feasible and may allow automated absolute quantification. The correlation with CT-morphology allows the exclusion of potentially false positive bone uptake which is not associated with the implant interface such as osteophytes, heterotopic calcifications and others. This was necessary in 25% of cases.

The comparison of diagnostic accuracies of the different quantification methods in our study showed that the combination of the xSPECT Bone with CT including iterative metal artefact reduction for attenuation correction performed best in diagnosing prosthetic loosening with an accuracy of 93.9%. Most likely, this can be explained with the higher resolution of the xSPECT Bone data compared to xSPECT Quant data reducing the partial volume effect, thus leading to a higher discrimination of different uptake levels.

Moreover, CT-metal artefact reduction with iMAR does not only improve the image quality of CT images for morphologic assessment, it also showed to improve attenuation correction around metal implants as shown in a recently published study [26]. CT-streak artefacts around the metal implant can influence the zone mapping of xSPECT bone and may lead to a false attribution of a tissue class to a voxel, as streaks artificially change the depicted density. A soft tissue may then be classified as “cortical bone” class and then obtain initially bone activity normalization. As iteration continues the activity estimate in that voxel is being reduced towards the correct soft tissue uptake, but iteration typically terminates well before then. What remains is an uptake which is higher than soft tissue, yet lower than actual bone tissue. These “shining artefacts” as described in a recent case series by Lima et al. [27] were not evident in our study; a reason may be the marked reduction of these artefacts with iMAR, which may lead to a more precise quantification and may have contributed to the high accuracy of the combination xSPECT bone with iMAR reconstructed CT in our study (Fig. 2 versus Fig. 5).

Visual reading of triple phase scintigraphies together with SPECT/CT showed a lower accuracy compared to quantitative uptake measures. Interestingly early phases did not contribute to the diagnostic decision with an accuracy of only 48% for early phases alone. The difference of accuracies of visual reading and quantitative uptake measures was, however, not significant. Nevertheless, quantification of periprosthetic uptake as proposed could add confidence to the diagnostic decision, especially for physicians with limited experience.

This study has several limitations: Due to the selection criteria, the study cohort may not be representative for the broader and more heterogeneous collective of patients with symptomatic prosthetic joints as seen in general practices and ambulant facilities. In particular, the pre-test probability may be lower compared to our cohort. As a consequence, the accuracy of quantitative SPECT/CT may be lower in this context. The small cohort size of 30 patients did not allow subgroup analysis such as knee versus hip joint prostheses or uncemented versus cemented prostheses. Still, we found highly significant differences with clearly higher uptake levels in loosened arthroplasties even in this small cohort.

We compared quantitative uptake values of four different reconstruction methods and compared several diagnostic parameters (accuracy, sensitivity, specificity, positive and negative predictive values) of these four quantification methods and the visual readings. This leads to the problem of multiple comparisons and the possibility that some of the significant differences could be produced by chance should be taken into account when interpreting our results. However, since the differences of SUVmax between loosened and stable prosthesis are very clear and highly significant and the diagnostic parameters, which are closely related to each other show a consistent tendency, we believe that the results are robust.

We included some patients (*n* = 4) with a postoperative time interval between 6 and 12 months. In this relatively early postoperative phase, in which implant failures can occur, a higher bone metabolism can be expected also in asymptomatic patients [28, 29]. A sensitivity analysis on the time interval between implantation and SPECT/CT did not reveal a significant influence on the results. Still in this early implantation phase periprosthetic uptake has to be interpreted with caution and SUV values may not be reliable for the diagnosis of aseptic loosening.

Also, the heterogeneity of implant models with cemented and uncemented arthroplasties might have biased the results, e.g. it was shown by Ullmark et al. that uncemented prostheses have higher and longer lasting ^18^F-fluoride uptake levels compared to cemented stems [30]. Again, we controlled for this bias performing a sensitivity analysis and did not find a significant influence on the results.

We did not analyse the distribution pattern of tracer uptake around implants as did other studies [16] since we wanted to evaluate [^99m^Tc]Tc-DPD uptake values as a quantitative biomarker for the diagnosis of prosthetic loosening independent of uptake patterns.

## Conclusion

All evaluated quantification methods showed a significantly higher [^99m^Tc]Tc-DPD uptake around loose arthroplasties than around stable arthroplasties. Importantly, quantification with xSPECT Bone plus iMAR discriminates best between loose and stable prostheses. With cut-off values derived by ROC analysis, an accuracy of nearly 94% could be reached solely with quantitative uptake measures proposing [^99m^Tc]Tc-DPD uptake quantification as a promising biomarker for the diagnosis of prosthetic loosening. However, further studies will be necessary which will analyse and quantify periprosthetic uptake pattern in a larger number of patients with symptomatic knee and hip prostheses in order to fully utilize the potential of uptake quantification as a biomarker.

## Data Availability

The datasets used and analysed during the current study are available from the corresponding author on reasonable request.
